# Network Support Using Social Networking Services to Increase Exercise Adherence Among Korean-Chinese Middle-Aged Migrant Women: Mixed Methods Study

**DOI:** 10.2196/19159

**Published:** 2020-11-05

**Authors:** Hyeyeon Lee, Hyeonkyeong Lee, Youlim Kim, Sookyung Kim, Young-Me Lee

**Affiliations:** 1 College of Nursing Yonsei University Seoul Republic of Korea; 2 Mo-Im Kim Nursing Research Institute College of Nursing Yonsei University Seoul Republic of Korea; 3 School of Nursing College of Science and Health Depaul University Chicago, IL United States

**Keywords:** SNS, social support, network support, exercise adherence, social-cognitive factors, text mining

## Abstract

**Background:**

Social networking services (SNSs) are recognized to be a promising approach to easily deliver health interventions and to enhance social support for exercise adherence. However, the patterns and aspects of social support through SNSs have not been reported and their influence on other social-cognitive factors remains inconclusive.

**Objective:**

Our objective is to explore how social support delivered through SNSs impacts interactions among Korean-Chinese (KC) middle-aged women and to identify how this approach influences social-cognitive factors for exercise (eg, sense of community, self-efficacy for exercise, and social support for exercise).

**Methods:**

A mixed methods design was used. Text analysis of SNS messages and text mining using the Korean Natural Language Application (KoALA) were conducted. Social-cognitive factors (eg, sense of community, self-efficacy for exercise, and social support for exercise) were assessed at baseline and after 12 weeks using a structured questionnaire. A comparison of social-cognitive factors at baseline and at 12 weeks was conducted to identify any potential significant changes, using the Wilcoxon signed-rank test.

**Results:**

A total of 259 SNS messages were collected from 24 KC women, distributed among four chat groups, who participated in a 12-week walking intervention program between August and October 2018. The individual average frequency of chatting via the SNS was 10.79 (range 0-34) and the most frequent type of social support through the SNS was network support (172/259, 66.4%). The most common words extracted from the SNS were *Health*, *Exercise*, *Participation*, and *We*. Overall, the perceived levels of sense of community (*P*<.001) and social support for exercise (*P*=.002) were significantly increased at 12 weeks compared with baseline. Group 1 (*P*=.03) and Group 4 (*P*=.03), whose members demonstrated the highest frequency of network support, experienced a significant increase only in the level of sense of community.

**Conclusions:**

By integrating these data and conducting a mixed methods analysis, we observed that among the types of social support, network support was a key point for the promotion of social-cognitive factors in increasing exercise adherence. Therefore, network support through SNS-based interventions should be considered as a useful strategy to help vulnerable migrant populations make changes to exercise behaviors.

## Introduction

### Social Networking Services as Useful Tools for Health Promotion

Interpersonal relationships and social interactions are shifting from face-to-face to online environments due to the development of the internet and smart devices, as well as an increase in economic development [1]. Social networking services (SNSs) have enabled individuals to interact and connect with others more easily, regardless of time and place, including those with diverse racial and ethnic backgrounds. While there are negative influences of SNSs on well-being due to social comparison and isolation [2], individuals easily establish new interpersonal networks and experience psychological well-being (eg, happiness and satisfaction with life) by using SNSs, such as Facebook or Twitter [3,4]. SNSs are also recognized as valuable and excellent platforms for delivering health interventions to hard-to-reach minority populations [4].

### Exercise and Social-Cognitive Factors in Migrant Populations

The population of South Korea is multicultural, with more than 2.1 million migrants in 2017, including roughly 680,000 (32.9%) Korean-Chinese (KC) [5]. The proportion of this KC population that is made up of middle-aged women (ages 40-65 years) is 33.17% (n=225,488), and these women often work in the restaurant service industry or as houseworkers [5,6]. Due to changes in their health, middle-aged women are vulnerable to physical and mental health problems (eg, cardiovascular disease, type 2 diabetes, obesity, hypertension, and depression) [7,8]. Although physical activities are important for managing the health of middle-aged women, migrant groups often have lower participation rates in programs that promote a healthy lifestyle compared to other groups, due to cultural differences, insufficient knowledge of health services, and limited access [9,10]; access may be considerably limited due to the working environment (eg, less flexible and less autonomous) compared with native Koreans [10]. These women face many other barriers, including physical-related barriers (eg, lack of time and space to exercise), knowledge-related barriers (eg, lack of knowledge about exercise), psychological barriers (eg, low self-efficacy), and lack of social support [11]. As a result, it is especially difficult to implement physical activity–based interventions for middle-aged migrant women.

As a strategy to encourage exercise adherence in migrant populations, social-cognitive factors such as social support have been commonly adopted in interventional designs [12]. Recently, SNS-based interventions emphasizing social support as well as social-cognitive factors have been found to increase exercise adherence. For instance, in one study of adults with type 2 diabetes, participants in a private Facebook group that received feedback, coaching, and social support in real time increased the number of steps taken in a day compared to those in the nonintervention group [13]. However, earlier studies only identified and categorized the perceived degree of social support through SNSs without an analysis of the patterns or aspects of social support during the intervention period [14,15]. In addition, although existing evidence demonstrates that social support has a positive correlation with other social-cognitive factors (eg, sense of community and self-efficacy for exercise) [16-18], there are still a limited number of studies, and results remain inconclusive.

### Definition and Types of Social Support

#### Overview

Social support is defined as interpersonal interactions that involve emotional attention, instrumental assistance, information about the environment, and a positive self-assessment [19,20]. Types of social support through SNSs in this study are based on previous studies and include the following: (1) network support, (2) emotional support, (3) information support, and (4) esteem support [19-21]. Exceptionally tangible or instrumental support was excluded from the analysis because it is based on private topics and offline conversations [22]. Additionally, gratitude is related to social support and substantially and positively affects emotional support; this study included the expression of gratitude as a subdomain of emotional support [23].

#### Network Support

Network support is defined as the presence of companions or potential aides (eg, “Find assistants to help you”) who share or engage in social activities and resources [2,21,24]. Network support is identified from the desire to be emotionally satisfied and to maintain a relationship with a group to which one belongs [25]. In the online health community, a network could include conversations about members’ interests [21] and life-related topics that are not related to health issues [22,26]. Subcategories of network support are access, presence, and companionship [20,27]. Access refers to a person in a similar situation asking questions on behalf of you or sharing mailing address and/or contact information. Presence involves a supportive expression indicating that you are willing to help others. Companionship includes messages that reassure members that they share a similar environment and experience, including experiences not related to health issues [28] (eg, nonverbal cues, such as emoticons, narratives, poetry, humor, jokes, and vacation planning).

#### Emotional Support

Emotional support includes expression, encouragement, sympathy, affection, empathy, care, and concern [14,22,26] (eg, “You can do it!” “I believe,” and “Keep it up”). Subcategories of emotional support are relationship, physical affection, confidentiality, sympathy, understanding or empathy, encouragement, prayer, and gratitude. Relationships are indicated with important messages that express friendliness or closeness within the online community. “Good morning” and “Nice to meet you” are often used as the first phrases of conversation conveying emotional support. Physical affection in an SNS is delivered as text-based messages (eg, “HUG”) and provides emotional support, such as physical stability. Confidentiality is related to protection of secrets, whereas sympathy is an expression of sorrow for another member’s stress and suffering. Understanding is an expression of empathy to another member who is experiencing difficult and/or complex situations. Encouragement includes messages that convey hope and confidence to other members. Prayer offers a blessing for other members, and gratitude is an expression of appreciation.

#### Information Support

Information support involves the delivery of information or the provision of guidelines or suggestions (eg, information about physical activity, recommendations, and how to deal with symptoms) [14,22,26]. Subcategories of information support include advice, referral, situational appraisal, and teaching. Referral involves sharing specific informational resources (eg, books, websites, and institutions) with other members. Situational appraisal might come in the form of a message to help re-evaluate another member’s situation. Teaching involves the dissemination of factual information.

#### Esteem Support

Esteem support involves sharing a complimentary expression, such as “Congratulations” or “Great job” [14,26]. Subcategories of esteem support are compliment, validation, and relieving of blame. A compliment is a celebration of accomplishment despite a negative situation or a positive statement to other members. Validation involves agreeing with the feelings and stories of other members or to support their view of the situation. Relieving of blame comes in the form of an expression of support for another member who expresses guilt for a particular situation.

### Purpose of This Study

The purpose of this study was to (1) explore how social support through SNSs appeared in interactions among KC middle-aged women who are vulnerable to health problems and (2) identify how this social support influenced social-cognitive factors for exercise (eg, sense of community, self-efficacy for exercise, and social support for exercise).

## Methods

### Design

The mixed methods design was used to combine qualitative and quantitative data to better understand patterns of social support and its effect on other social-cognitive factors potentially impacting exercise adherence.

### Participants and Data Collection

A community-based project was conducted to improve physical and mental health of KC migrant women through a 6-month, culturally adaptive, mobile app–based walking intervention between May 2017 and February 2019 [29]. For recruiting participants, we visited a public health center, the Korea Support Center for Foreign Workers, and a KC church; distributed leaflets; and promoted the health intervention program. Participants in the study were KC middle-aged women (40-65 years of age) who had no health restrictions against increasing physical activity. Based on physical activity assessed using the Physical Activity Readiness Questionnaire, those who walked more than three times a week and more than 30 minutes at a time were excluded. Only those who were able to use the smartphone app and participate in the program for 6 months were included in the study. There were 28 participants in the experimental group, all of whom completed the baseline assessment after completing the informed consent form. In order to promote social support and networking for physical activity, these 28 KC middle-aged women were divided into four groups. A group leader was appointed by vote within each group and instructed to encourage and support other group members throughout the study to increase exercise adherence.

Data collection was conducted over an intervention period of 12 weeks between August and October 2018. Out of the 28 participants, 1 (4%) dropped out at the fifth week and 2 (7%) did not complete the 12-week assessment and were, thus, excluded from analysis. During the 12-week intervention period, a total of 353 messages were collected via KakaoTalk, the most common chatting app in Korea. However, a woman whose comments made up a disproportionate number of total SNS conversations (94/353, 26.6%) was excluded because her contributions may have distorted the study results. Ultimately, all 259 text messages written in Korean from the SNS groups of 24 KC middle-aged women were used in the final analysis of this study.

### Qualitative Data Analysis: Text Analysis and Text Mining

#### Text Analysis

Text analysis was conducted on 259 messages sent among the 24 participants via SNS over the 12 weeks by types of social support. Two researchers (Hyeyeon L and YK) independently coded all 259 messages according to social support codes. Inconsistent codes were reviewed and discussed with the principal investigator (Hyeonkyeong L) to reach consensus [14].

#### Text Mining

Text mining was conducted using the Korean Natural Language Application (KoALA) (Mondata Co) to identify themes and interests of KC middle-aged women interacting using an SNS. The KoALA engine is an application for social media mining and supports the entire text-mining process, including social media data collection, preprocessing, and analysis [30]. A word cloud visually highlights keywords based on how frequently they are used. Co-occurrence analysis is a matrix form that calculates the weight based on the frequency of keywords appearing in the text at the same time. The simultaneous appearance of certain keywords in the text means that they are related to each other, and the repetition of these keywords indicates a high correlation between the keywords.

Four chat room conversations undertaken over 12 weeks using an SNS were integrated. After extracting all text and dividing it into sentence units, the process of recognizing entity names was conducted using the entity name dictionary. The next step was to remove stop words. Data analysis was performed using the word cloud approach and co-occurrence matrix. After preprocessing was complete, 20 nouns were extracted from the chat group in order of appearance. Those were used to analyze the word *exercise* and the words that appeared as co-occurring terms.

### Quantitative Data Analysis

#### Overview

Social-cognitive factors (eg, sense of community, self-efficacy for exercise, and social support for exercise) were assessed at baseline and at 12 weeks. General characteristics, mean, standard deviation, and frequency of data were analyzed by descriptive statistics using SPSS Statistics for Windows, version 25.0 (IBM Corp). Additionally, the Wilcoxon signed-rank test was conducted to confirm the significance of sense of community, self-efficacy for exercise, and social support for exercise by individuals and groups.

#### Sense of Community

Sense of community was measured using the Korean version of the Brief Sense of Community Scale, a tool based on the scale developed by Peterson et al [31] and later modified by Oh [32]. The scale involves eight items rated on a 5-point Likert scale, ranging from 1 (strongly disagree) to 5 (strongly agree). A higher score represents a greater sense of community. Among the items, *peer/friend* was changed to *neighbor*, and *peer group* was changed to *neighborhood*. In the study, the Cronbach alpha was .73.

#### Self-Efficacy for Exercise

Self-efficacy for exercise was measured using the Korean version of the Barrier Self-Efficacy Scale developed by McAuley [33] and modified by Choi [34]. The scale includes 13 items, and ratings range from 0 (completely certain that I could not) to 10 (completely certain that I could). The higher the average score, the higher the self-efficacy for exercise. In the study, the Cronbach alpha was .89.

#### Social Support for Exercise

Social support for exercise was assessed using a questionnaire developed by Sallis et al [35] and translated and modified by Choi [36]. In this study, six items were selected, except the one added by Choi. The scale includes six items that assess perceived social support for exercise from family and friends during the past 3 months. The scale is rated using a 4-point Likert scale, ranging from 1 (strongly disagree) to 4 (strongly agree). The higher the total score, the higher the social support from family and friends. In the study, the Cronbach alpha was .88.

## Results

### Participant Characteristics

A total of 24 KC middle-aged women participated in the SNS groups. The average age of participants was 47.2 (SD 6.5) years; average time spent working daily was 10 (SD 4) hours; average duration of stay in Korea was 144.2 (SD 79.1) months (about 12 years); average number of months working at current job was 39.6 (SD 50.3); and average monthly income was about US $1504.50 (SD 469.75). Most participants had an educational level above high school graduate (17/24, 71%), and the service industry (ie, cook, waiter, housekeeper, hairdresser, and caregiver) was the most common job type (10/24, 42%). Moreover, 259 text messages written in Korean from the SNS groups were collected over an intervention period of 12 weeks between August and October 2018. The individual average frequency of chats via SNS was 10.79 (range 0-34).

### Qualitative Findings

#### Text Analysis: Social Support in the SNS

Social support in this study was divided into 18 subcategories (see Table 1). The most frequent type of social support through the SNS identified by this group was network support (172/259, 66.4%), followed by emotional support (40/259, 15.4%), information support (28/259, 10.8%), and esteem support (19/259, 7.3%).

**Table 1:** Conceptual definitions of social support and frequency of messages of each type.

Categories and subcategories of social support		Frequency of messages (N=259), n (%)	
**Network support**			
	Total		172 (66.4)
	1. Access		0 (0)
	2. Presence		0 (0)
	3. Companionship		172 (66.4)
**Emotional support**			
	Total		40 (15.4)
	4. Relationship		11 (4.2)
	5. Physical or visual affection		2 (0.8)
	6. Confidentiality		0 (0)
	7. Sympathy		1 (0.4)
	8. Understanding or empathy		0 (0)
	9. Encouragement		14 (5.4)
	10. Prayer		0 (0)
	11. Gratitude		12 (4.6)
**Information support**			
	Total		28 (10.8)
	12. Advice		10 (3.8)
	13. Referral		0 (0)
	14. Situational appraisal		1 (0.4)
	15. Teaching		17 (6.6)
**Esteem support**			
	Total		19 (7.3)
	16. Compliment		13 (5.0)
	17. Validation		6 (2.3)
	18. Relieving of blame		0 (0)

In total, 66.4% (172/259) of the support messages collected from the SNS were network support messages. Network support messages made up three of the 18 subcategories of social support; of these three subcategories, all messages involved companionship (172/172, 100%). Companionship includes a response, for example, to attending an offline cultural class, naming a group, talking about daily life, and discussion about exercise barriers; companionship may come in the form of short opinions, photos, and emoticons (eg, 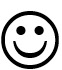 and 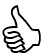). No messages corresponded to access or presence.

Emotional support messages within the SNS made up 15.4% (40/259) of all support messages and eight of the 18 subcategories of social support. In this study, all emotional support messages fell into one of five of these eight subcategories: (1) encouragement (14/259, 5.4%), (2) gratitude (12/259, 4.6%), (3) relationship (11/259, 4.2%), (4) physical affection (2/259, 0.8%), and (5) sympathy (1/259, 0.4%). No messages corresponding to confidentiality, understanding or empathy, or prayer were noted.

Information support messages within the SNS made up 10.8% (28/259) of all support messages and four of the 18 social support subcategories. In this study, all information support messages fell into one of three of these four subcategories: (1) teaching (17/259, 6.6%), (2) advice (10/259, 3.8%), and (3) situational appraisal (1/259, 0.4%). No referral messages were observed in this study.

Esteem support messages obtained from the SNS accounted for 7.3% (19/259) of all messages. These esteem support messages are categorized into two out of three of the 18 subcategories of social support: (1) compliment (13/259, 5.0%) and (2) validation (6/259, 2.3%). No messages from this study were categorized as relieving of blame. Example quotes by the type of social support are addressed in Table 2.

**Table 2:** Example quotes from messages of each type of social support.

Type of support		Quote	
**Network support: companionship**			
	Offline culture class	“It is difficult for me to attend.” [ID 5, September 9, Group 1] “Ok, I will attend.” [ID 9, August 15, Group 1] “Where will it be held?” [ID 2, September 4, Group 1]	
	Naming a group	“Beautiful Women Club?” [ID 45, August 17, Group 4] “How about Happy Club?” [ID 26, August 10, Group 3] “I’d like to recommend ‘Happy Club’ too; I think if we are not happy, our health is meaningless.” [ID 28, August 14, Group 3]	
	Daily life	“I’m on vacation for 5 days.” [ID 2, August 21, Group 1] “I’ll have salad for breakfast.” [ID 2, September 14, Group 1] “What sauce?” [ID 5, September 14, Group 1]	
	Exercise barriers	“I do well walking, but muscle exercise is difficult for me.” [ID 20, August 10, Group 2] “It is hard to keep myself motivated for doing muscle exercise.” [ID 18, August 10, Group 2] “I can’t exercise because the weather is hot.” [ID 25, August 10, Group 2]	
**Emotional support**			
	Relationship	“Nice to meet you.” [ID 22, August 10, Group 2] “Have a nice day.” [ID 2, September 17, Group 1] “Have a nice weekend.” [ID 26, September 29, Group 4]	
	Physical affection	“Emoticon: kiss.” [ID 2, August 29, Group 1]	
	Sympathy	“I’m sorry to hear that.” [ID 26, September 4, Group 3]	
	Encouragement	“Let's exercise this week as well for health.” [ID 43, August 23, Group 4]	
	Gratitude	“Thanks everyone.” [ID 26, August 23, Group 3]	
**Information support**			
	Advice	“To eat less sodium, I put some vegetables in Korean noodles.” [ID 19, September 18, Group 3]	
	Situational appraisal	“This offline cultural class is an essential education for a healthy life, I think.” [ID 26, September 4, Group 3]	
	Teaching	“The phone will work again if you turn the phone off and on.” [ID 18, August 13, Group 2] “It is good to leave three holes of band to wear the Fitbit band.” [ID 22, August 10, Group 2]	
**Esteem support**			
	Compliment	“Congratulations.” [ID 2, August 25, Group 1] “The yellow umbrella and your elegant fashion go well together; it's so pretty.” [ID 26, August 28, Group 2]	
	Validation	“I agree.” [ID 28, August 10, Group 3] “When you put on makeup after taking a makeup class, your face definitely looks better.” [ID 28, August 20, Group 3]	

#### Text-Mining Findings

**Figure 1:**
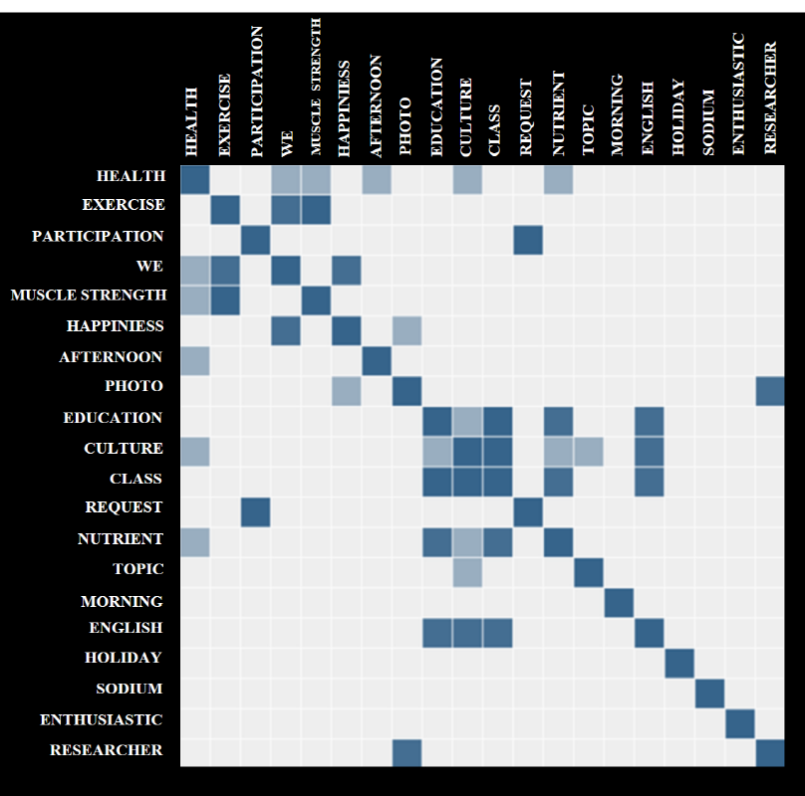
Co-occurrence matrix. The darker the color of the square, the more frequently the pair of words appears.

A keyword-extraction algorithm was used to identify the top 20 keywords and confirm whether these keywords were related to the exercise-adherence program analyzed here. The most common words extracted from the SNS were *Health* (n=21), *Exercise* (n=20), *Participation* (n=18), and *We* (n=18). The keyword used the most was *Health*, which confirmed that participants mainly talked about health-related topics on the SNS. The second-most used keyword was *Exercise*. Participants talked about their compliance with muscular exercise and walking related to *Exercise*. The third-most used keywords were *Participation* and *We*, confirming that these 24 KC middle-aged women encouraged one another to participate in exercise, referring to themselves as “We” with the goal of exercise adherence. As a result of co-occurrence analysis, the words that appeared to be highly related to *Health* were *Exercise*, *We*, *Muscle strength*, and *Happiness* (see Figure 1). It was found that KC middle-aged women encouraged *Exercise* and formed a sense of belonging of *We* regarding health in the SNS. The words *We* and *Happiness* were highly related, and the words that appeared most often with *Culture* were *Education* and *Class*.

### Quantitative Findings

#### Changes in the Levels of Social-Cognitive Factors at Baseline and at 12 Weeks

The Wilcoxon signed-rank test was conducted to analyze changes in sense of community, self-efficacy for exercise, and social support for exercise among KC middle-aged women from baseline to 12 weeks. Overall, the perceived levels of sense of community (*z*=–3.30, *P*<.001) and social support for exercise (*z*=–3.09, *P*=.002) were significantly increased at 12 weeks compared with baseline (see Table 3). The level of sense of community after the 12-week intervention increased significantly only in Group 1 (*z*=–2.20, *P*=.03) and Group 4 (*z*=–2.20, *P*=.03). The only increase in self-efficacy for exercise was in Group 1 (*z*=–2.03, *P*=.04), and the only increase in social support for exercise was in Group 4 (*z*=–2.02, *P*=.04).

**Table 3:** Changes in the levels of social-cognitive factors at baseline and 12 weeks.

Factors			Total (N=24)				Group 1 (n=6)				Group 2 (n=8)				Group 3 (n=4)				Group 4 (n=6)				
			Mean (SD)		*P* value^a^		Mean (SD)		*P* value^a^		Mean (SD)		*P* value^a^		Mean (SD)		*P* value^a^		Mean (SD)		*P* value^a^		
**Sense of community^b^**																							
	Baseline	23.79 (7.35)		*<.001* ^c^		24.17 (3.66)		*.03*		21.75 (8.71)		.80		18.75 (7.46)		.07		29.50 (5.39)		*.03*			
	12 weeks	29.25 (8.24)				31.17 (4.02)				22.00 (7.60)				28.00 (7.35)				37.83 (2.14)					
**Self-efficacy for exercise^d^**																							
	Baseline	6.92 (1.95)		.18		6.17 (2.32)		*.04*		6.75 (1.58)		.23		8.00 (2.00)		.71		7.17 (2.14)		.07			
	12 weeks	7.46 (2.30)				8.67 (1.21)				5.63 (2.67)				7.75 (0.50)				8.50 (2.07)					
**Social support for exercise^e^**																							
	Baseline	14.83 (5.22)		*.002*		17.00 (3.63)		.21		13.13 (3.83)		.12		15.00 (7.75)		.29		14.83 (6.74)		*.04*			
	12 weeks	18.63 (4.17)				19.50 (2.59)				15.00 (4.01)				20.50 (1.92)				21.33 (3.78)					

^a^The Wilcoxon signed-rank test was conducted.

^b^Measured using the Korean version of the Brief Sense of Community Scale; scores range from 1 (strongly disagree) to 5 (strongly agree).

^c^Italicized *P* values indicate significant results.

^d^Measured using the Korean version of the Barrier Self-Efficacy Scale; scores ranged from 0 (completely certain that I could not) to 10 (completely certain that I could).

^e^Assessed using a questionnaire developed by Sallis et al [35] and translated and modified by Choi [36]; scores ranged from 1 (strongly disagree) to 4 (strongly agree).

#### The Frequency of Each Type of Social Support Message Provided by Each Group

After analyzing the social support pattern and frequency by group, the frequency of network support was found to be higher than other types of social support in all groups. When comparing the social support frequency by group, Groups 1 and 4 demonstrated the highest network support frequency of over 70% (see Table 4). Due to the limited amount of data, the correlation and significance among types of social support and social-cognitive factors could not be studied.

**Table 4:** The frequency of each type of social support message provided by each group.

Group	Social support messages by type (N=259), n (%)				
	Network	Emotional	Information	Esteem	Total
1	56 (73.7)	10 (13.2)	3 (3.9)	7 (9.2)	76 (29.3)
2	33 (62.3)	5 (9.4)	14 (26.4)	1 (1.9)	53 (20.5)
3	35 (48.6)	18 (25.0)	11 (15.3)	8 (11.1)	72 (27.8)
4	48 (82.7)	7 (12.1)	0 (0)	3 (5.2)	58 (22.4)
Total	172 (66.4)	40 (15.4)	28 (10.8)	19 (7.4)	259 (100)

## Discussion

### Principal Findings

The main result of this study is that the most frequent type of social support through the SNS was network support and that levels of social-cognitive factors, including sense of community, self-efficacy for exercise, and social support for exercise, increased in groups with a high frequency of network support. Moreover, the simultaneously appearing keywords were *We*, *Exercise*, and *Happiness*. This study analyzed SNS messages shared among KC middle-aged women who participated in mobile app–based health promotion programs to examine the patterns of social support and to identify keywords and their associations through text mining. In addition, through intervention, changes in the levels of social-cognitive factors among KC women were confirmed through statistical analysis. To achieve this, SNS messages exchanged among group members for 12 weeks were characterized using text analysis, text mining, and statistical methods. It is worth mentioning that, to our knowledge, this is the first attempted analysis of SNS text messages to examine patterns of social support.

### Strengths

This study has a number of strengths. First, the result of the mixed methods analysis demonstrated that the most common type of social support conveyed through SNS messages was network support, followed by emotional support, information support, and esteem support. This finding is incongruent with other studies that have examined the influence of social support through an SNS. In a study of an online community for breast cancer survivors, the most common social support identified was information support [22]. In another intervention designed to improve smoking cessation efforts, information and emotional support were found to be the most frequent supports [37]. Network support includes not only stories from the everyday lives of the health-related online community’s participants, such as chat, humor, and teasing (eg, birthday wishes and vacation plans), but also stories that are not related to health problems [22,26]. KC women often interacted through the SNS because they worked as house caretakers during the week throughout the intervention. Since KC women are often in similar situations and environments, it is thought that there is a high frequency of network support interactions like real-life-related topics between participants [38]. Thus, to approach migrant populations within this environment, network support can be hypothesized to impact health promotion [39].

Second, we found that levels of social-cognitive factors after a 12-week intervention were significantly increased in groups that had a high frequency of network support. In a previous study that analyzed dialogue between Canadian Indigenous women who participated in internet chat rooms [40], those who received social support through SNS activities were encouraged to enhance their confidence and self-efficacy for health promotion. Also, network support in an online community may help to solidify the social network and increase a sense of community among group members [28]. In another study that analyzed conversations on social media sites like Facebook, text messages related to network support derived more reciprocal responses among participants, and that study suggested that (1) network support involves communication with many other people with similar experiences and (2) there may be evidence that network support increases a sense of belonging to a social group [21,41]. This explanation is applicable to the findings of this study because the group with the most frequent network support experienced a significant increase in sense of community after 12 weeks. As in previous studies, it was shown to be necessary to increase social-cognitive factors for exercise adherence; social support facilitated an increase in social-cognitive factors [12,13]. Therefore, strengthening network support through an SNS in an online community may be useful for increasing physical activity.

Third, this study was the first to attempt text mining to analyze text messages shared via SNS among KC women. Quantitative analysis has limitations when trying to explore the real-life social network of participants. Through text mining, this study extracted interests, themes, and relationships of words using scientific evidence. In a study of alcohol and marijuana use, online text message analysis using text mining was shown to improve understanding of the participants’ impact on health-related behaviors and the role of social networks [42]. Also, by using text mining to analyze patterns of participants’ chats about electronic cigarette cessation, a study attempted to find factors of interest in developing strategies for promoting future behavioral change [43]. Specifically, co-occurrence word analysis confirmed the development process and structural relationships of scientific knowledge by extracting research topics and discovering the relationships among them [44,45].

After identifying the most common keywords (from #1 to #20) using a keyword-extraction algorithm, it was noted that KC middle-aged women frequently talked about exercise. This study confirmed that the use of an SNS encouraged participants to keep exercising because exercise was perceived as routine or a habit rather than as an extra burden [46]. Although it has been suggested that more evidence is needed to determine the actual usability of an SNS for changing behavior and increasing engagement [47], this study suggests that network support through an SNS may be an effective strategy for exercise adherence. Also, KC middle-aged women mainly talked about the contents of offline cultural classes addressing acculturation. Even if programs for cultural adaptation were provided to immigrants, low participation rates and high attrition rates would be likely be obstacles, as has been noted [46,48]. Although immigrants lack social networks and social support due to language or cultural differences, it was found that SNS-based group intervention can be a useful tool for enhancing the intercultural interaction among immigrants [48].

Moreover, co-occurrence analysis revealed which keywords were related to awareness of the promotion of physical activity. Specifically, *We*, *Exercise*, and *Happiness* were the most closely connected terms; the use of *We* in the chat group indicated that participants often thought collectively, and *We – Exercise* means that they encouraged one another to exercise and be healthy and perceived each other as a community. Another mobile phone–based intervention showed consistent findings through a walking group or buddies to enhance social support for exercise [49]. Moreover, the words *We* and *Happiness* were highly related, implying that SNS-mediated social support created a network of support among group members, leading to happiness in life as well as a form of exercise adherence. Therefore, an SNS-based group intervention is expected to be effective in forming network support and a sense of community and to promote health through exercise adherence for groups with limited access to health care. Studies that determine if network support can predict health outcomes such as happiness are apparently warranted.

### Limitations

Text analysis of SNS messages has several limitations; therefore, care is needed when interpreting the implications of these findings. First, some messages contain two or more types of social support. Since this study set the criteria for dialogue as the *send* button, within one dialogue phrase there can be examples of mixed social support. For example, a greeting, which corresponds with *emotional support*, and teaching, which corresponds with *information support*, can coexist in one dialogue message. Second, an additional type of social support might be needed. In this study, we identified a pattern for asking questions and finding answers in the SNS. Although this study classifies this conversation type as *network support*, it may also be further broken down into *seeking support* and *providing support *[22]. Third, there was a lack of data saturation. A total of 259 SNS text messages collected over 12 weeks was insufficient for text mining. Although the KoALA program, which is suitable for the Korean language, was used, 79 conversations included emoticons or URL video links and, thus, were excluded from text mining. As emoticons enrich the information provided on SNSs and strengthen social connections, further research to analyze emoticons is required, which will enhance usability [50-52]. Lastly, participation patterns in chats differed by group, and although positive effects through the SNS may occur, the potential for negative consequences (eg, loneliness and social comparison) cannot be excluded [47]. Therefore, further research must examine the potential negative influences of participation in SNSs and must suggest strategies to maximize the likelihood of positive outcomes following the use of an SNS.

### Conclusions

SNSs have been found to be essential communication tools for KC migrant women in Korea. The findings of this study improve our understanding of the potential impact of SNSs on improving social-cognitive factors related to exercise and promoting healthy behaviors in migrants. Specifically, the KC migrant women who participated in the culturally adaptive walking intervention described herein received network support from group members through the SNS. The group network support appeared to be more effective at increasing the levels of social-cognitive factors, which are known to be facilitators of healthy behaviors and acculturation, compared with other types of social support. Therefore, interventions designed to enhance network social support through SNSs may be effective strategies for improving health-related behaviors and cultural adaptation for migrant populations.

## Abbreviations

KC: Korean-Chinese
KoALA: Korean Natural Language Application
NRF: National Research Foundation
SNS: social networking service

## References

[1] Shepherd A, Sanders C, Doyle M, Shaw J (2015). Using social media for support and feedback by mental health service users: Thematic analysis of a twitter conversation. BMC Psychiatry.

[2] Xu Y, Burleson BR (2001). Effects of sex, culture, and support type on perceptions of spousal social support: An assessment of the "support gap" hypothesis in early marriage. Hum Commun Res.

[3] Erfani SS, Blount Y, Abedin B (2016). The influence of health-specific social network site use on the psychological well-being of cancer-affected people. J Am Med Inform Assoc.

[4] Park BK, Calamaro C (2013). A systematic review of social networking sites: Innovative platforms for health research targeting adolescents and young adults. J Nurs Scholarsh.

[5] (2017). Korea Immigration Service Statistics 2017.

[6] Piao Z (2013). A Study on the Health Status Among Middle-Aged Korean-Chinese Women Workers in Korea [master's thesis].

[7] Seifi B, Ghanizadeh G, Seyedin H (2018). Disaster health literacy of middle-aged women. J Menopausal Med.

[8] Chee W, Kim S, Chu T, Tsai H, Ji X, Zhang J, Chee E, Im E (2016). Practical issues in developing a culturally tailored physical activity promotion program for Chinese and Korean American midlife women: A pilot study. J Med Internet Res.

[9] Nerhus M, Berg AO, Haram M, Kvitland LR, Andreassen OA, Melle I (2015). Migrant background and ethnic minority status as predictors for duration of untreated psychosis. Early Interv Psychiatry.

[10] Lee H, Chae D, Lee K, Lee M (2013). Experiences of middle-aged Korean-Chinese female migrant workers in Korea: With focus on risk factors in work-related musculoskeletal diseases. J Korean Acad Community Health Nurs.

[11] Lee H, Wilbur J, Chae D, Lee K, Lee M (2015). Barriers to performing stretching exercises among Korean-Chinese female migrant workers in Korea. Public Health Nurs.

[12] Cooper LB, Mentz RJ, Sun J, Schulte PJ, Fleg JL, Cooper LS, Piña IL, Leifer ES, Kraus WE, Whellan DJ, Keteyian SJ, O'Connor CM (2015). Psychosocial factors, exercise adherence, and outcomes in heart failure patients: Insights from heart failure: A controlled trial investigating outcomes of exercise training (HF-ACTION). Circ Heart Fail.

[13] Bender MS, Cooper BA, Park LG, Padash S, Arai S (2017). A feasible and efficacious mobile-phone based lifestyle intervention for Filipino Americans with type 2 diabetes: Randomized controlled trial. JMIR Diabetes.

[14] Stragier J, Mechant P, De Marez L, Cardon G (2018). Computer-mediated social support for physical activity: A content analysis. Health Educ Behav.

[15] Park A, Conway M, Chen AT (2018). Examining thematic similarity, difference, and membership in three online mental health communities from Reddit: A text mining and visualization approach. Comput Human Behav.

[16] Chair SY, Wong KB, Tang JY, Wang Q, Cheng HY (2015). Social support as a predictor of diet and exercise self-efficacy in patients with coronary artery disease. Contemp Nurse.

[17] Lee D, Young SJ (2018). Investigating the effects of behavioral change, social support, and self-efficacy in physical activity in a collectivistic culture: Application of stages of motivational readiness for change in Korean young adults. Prev Med Rep.

[18] Tang F, Chi I, Dong X (2017). The relationship of social engagement and social support with sense of community. J Gerontol A Biol Sci Med Sci.

[19] House JS (1983). Work Stress and Social Support.

[20] Cohen S, Syme S (1985). Social Support and Health.

[21] Cutrona CE, Suhr JA (2016). Controllability of stressful events and satisfaction with spouse support behaviors. Communic Res.

[22] Wang X, Zhao K, Street N (2017). Analyzing and predicting user participations in online health communities: A social support perspective. J Med Internet Res.

[23] Fujitani T, Ohara K, Kouda K, Mase T, Miyawaki C, Momoi K, Okita Y, Furutani M, Nakamura H (2017). Association of social support with gratitude and sense of coherence in Japanese young women: A cross-sectional study. Psychol Res Behav Manag.

[24] Lin N, Simeone R, Ensel W, Kuo W (1979). Social support, stressful life events, and illness: A model and an empirical test. J Health Soc Behav.

[25] O'Reilly CA, Chatman J (1986). Organizational commitment and psychological attachment: The effects of compliance, identification, and internalization on prosocial behavior. J Appl Psychol.

[26] Braithwaite DO, Waldron VR, Finn J (1999). Communication of social support in computer-mediated groups for people with disabilities. Health Commun.

[27] Cobb S (1976). Presidential Address-1976. Social support as a moderator of life stress. Psychosom Med.

[28] Wang X, Zuo Z, Zhao K (2015). The evolution and diffusion of user roles in online health communities: A social support perspective. Proceedings of the 3rd International Conference on Healthcare Informatics (ICHI 2015).

[29] Kim Y, Lee H, Lee MK, Lee H, Jang H (2020). Development of a living lab for a mobile-based health program for Korean-Chinese working women in South Korea: Mixed methods study. JMIR Mhealth Uhealth.

[30] Jeon BJ, Choi YJ, Kim HW (2019). Application development for text mining: KoALA. Inf Syst Rev.

[31] Peterson NA, Speer PW, McMillan DW (2007). Validation of a Brief Sense of Community Scale: Confirmation of the principal theory of sense of community. J Community Psychol.

[32] Oh J, Kim C, Ha M (2012). A study on effect of adolescents' leisure satisfaction on self-esteem and sense of community: Focused on vocational high school students. Korea J Hosp Res.

[33] McAuley E, Courneya KS (1992). Self-efficacy relationships with affective and exertion responses to exercise. J Appl Soc Psychol.

[34] Choi M, Kim H (2008). Nutrition knowledge, dietary self-efficacy and eating habits according to student's stage of regular breakfast or exercise [article in Korean]. Korean J Community Nutr.

[35] Sallis JF, Grossman RM, Pinski RB, Patterson TL, Nader PR (1987). The development of scales to measure social support for diet and exercise behaviors. Prev Med.

[36] Choi J (2008). Construction of leisure physical activity model of middle-aged women in urban area. Korean J Adult Nurs.

[37] Granado-Font E, Ferré-Grau C, Rey-Reñones C, Pons-Vigués M, Pujol Ribera E, Berenguera A, Barrera-Uriarte ML, Basora J, Valverde-Trillo A, Duch J, Flores-Mateo G (2018). Coping strategies and social support in a mobile phone chat app designed to support smoking cessation: Qualitative analysis. JMIR Mhealth Uhealth.

[38] Coulson NS, Buchanan H, Aubeeluck A (2007). Social support in cyberspace: A content analysis of communication within a Huntington's disease online support group. Patient Educ Couns.

[39] Frohlich DO (2014). The social support model for people with chronic health conditions: A proposal for future research. Soc Theory Health.

[40] Wright K (2016). Social networks, interpersonal social support, and health outcomes: A health communication perspective. Front Commun.

[41] Andalibi N, Haimson OL, Choudhury MD, Forte A (2018). Social support, reciprocity, and anonymity in responses to sexual abuse disclosures on social media. ACM Trans Comput Hum Interact.

[42] Pizzicato LN, Barbour RC, Kershaw T (2019). Evaluating alcohol and marijuana use among emerging adult males via analysis of text messages. Subst Use Misuse.

[43] Chen AT, Zhu S, Conway M (2015). What online communities can tell us about electronic cigarettes and hookah use: A study using text mining and visualization techniques. J Med Internet Res.

[44] He Q (1999). Knowledge discovery through co-word analysis. Libr Trends.

[45] Small H (1999). Visualizing science by citation mapping. J Am Soc Inf Sci.

[46] Laranjo L, Neves AL, Costa A, Ribeiro RT, Couto L, Sá AB (2015). Facilitators, barriers and expectations in the self-management of type 2 diabetes--A qualitative study from Portugal. Eur J Gen Pract.

[47] Balatsoukas P, Kennedy CM, Buchan I, Powell J, Ainsworth J (2015). The role of social network technologies in online health promotion: A narrative review of theoretical and empirical factors influencing intervention effectiveness. J Med Internet Res.

[48] Erdem B (2018). US-Turkey relations in the light of Turkey's freedom of press. Glob Media J.

[49] Lindgren T, Hooper J, Fukuoka Y (2019). Perceptions and experiences of women participating in a digital technology-based physical activity intervention (the mPED Trial): Qualitative study. JMIR Public Health Surveill.

[50] Cui M, Jin Y, Kwon O (2016). A method of analyzing sentiment polarity of multilingual social media: A case of Korean-Chinese languages [article in Korean]. J Intell Inf Syst.

[51] Edo-Osagie O, Smith G, Lake I, Edeghere O, De La Iglesia B (2019). Twitter mining using semi-supervised classification for relevance filtering in syndromic surveillance. PLoS One.

[52] Hsieh SH, Tseng TH (2017). Playfulness in mobile instant messaging: Examining the influence of emoticons and text messaging on social interaction. Comput Human Behav.

